# Can observational learning reinforce open-label placebo hypoalgesia?

**DOI:** 10.1097/j.pain.0000000000003161

**Published:** 2024-01-16

**Authors:** Justyna Brączyk, Przemysław Bąbel

**Affiliations:** a Jagiellonian University, Institute of Psychology, Pain Research Group, Kraków, Poland; b Doctoral School in the Social Sciences, Jagiellonian University, Kraków, Poland

**Keywords:** Open-label placebo, Placebo hypoalgesia, Placebo effect, Verbal suggestion, Observational learning

## Abstract

Previous research has indicated that an open-label placebo can reduce pain in both healthy participants and patients with chronic pain. Because nondeceptive placebos seem to be an effective and more ethical alternative to deceptive placebos, optimizing this kind of treatment is essential. Observational learning was previously shown to induce the deceptive placebo effect; therefore, this study aimed to verify its effectiveness in fortifying the open-label placebo effect. Healthy volunteers (N = 117) were randomly assigned to 4 groups: open-label placebo with observational learning (OLP + OBL), open-label placebo (OLP), deceptive placebo with observational learning (OBL), or control group. Participants underwent baseline and testing measurements, during which they self-reported pain induced by heat stimulation. Between assessments, placebo cream was openly administered in the OLP and OLP + OBL groups. The OLP + OBL group next watched a model experiencing hypoalgesia after cream application. In the OBL group, participants received placebo cream with no information about its effect, and then they watched the model. The placebo effect was successfully evoked in all experimental groups (OLP + OBL, OLP, and OBL), which confirms the effectiveness of both open-label and deceptive placebo interventions for pain reduction. However, the hypoalgesic effect was of similar magnitude in the OLP and OLP + OBL groups, which indicates that observation did not contribute to the effect. The results showed that reinforcing the open-label placebo by observational learning may be redundant, but more research is needed to confirm these findings.

## Abstract 2

The open-label placebo effect seems to be effective in reducing pain. Adding observational learning to the open-label procedure to reinforce the effect may be redundant.

## 1. Introduction

Although placebo treatment seems to be effective^23,36,48^ and legitimate under certain conditions,^9,43^ it is often considered unethical.^16,24^ One of the main complaints is that placebos require deception, leading to a lack of awareness by patients regarding the exact treatment and making it impossible to obtain fully informed consent. Despite this concern, placebos are still readily administered by medical staff^2,21,37,47^ because they find placebo useful and beneficial for patients.^21^ Therefore, it seems imperative to find the most sound approach for using inactive treatment.

Open-label placebo entails the transparent and honest administration of an inactive substance, without any deception. To render a placebo treatment open, researchers frequently provide a scientific rationale concerning the mechanisms of placebo effects and their effectiveness.^27–29,31^ To date, several studies have indicated that nondeceptive placebos may help people manage a variety of clinical disorders and nonclinical impairments, including chronic pain.^12,13,27,33^ Meta-analyses^14,52^ have shown that open-label placebo can exert a medium effect size, effectively reducing various medical symptoms. Furthermore, the effectiveness of open placebos was comparable with that of deceptive placebos.^19,38,40^

The deceptive placebo effect in pain has effectively been evoked by verbal suggestion and several learning processes, including observational learning. Verbally provided suggestion is the most established method of producing placebo hypoalgesia. It involves raising specific expectations concerning pain intensity by describing the potential impact of the placebo intervention to the patient.^6,34,41,46^ The verbal suggestion also lies at the core of the open-label placebo effect.^8,28^ Furthermore, previous studies on deceptive placebo effects have shown that observing another person experience decreased pain after placebo use caused corresponding changes in participants' pain intensity ratings.^7,10,15,20,25,39,45^ The placebo effects resulting from observational learning are believed to be rooted in the acquisition of specific expectations.^17,32^ Moreover, modeling of placebo effects was shown to be effective both when the model's behavior is observed directly^5,15,44^ or presented in a video recording,^7,10,49,50^ and the effectiveness of these 2 methods was comparable.^25^

To our knowledge, no studies have examined the effectiveness of observational learning in inducing the open-label placebo effect. This study was aimed to induce (1) the open-label placebo effect by verbal suggestion and observation, (2) the open-label placebo effect by verbal suggestion alone, and (3) the deceptive placebo effect by observation. We hypothesized that the placebo effect will be successfully induced by all these methods (H1). Moreover, previous research on the deceptive placebo effect showed that observational learning combined with verbal suggestion^15,25^ elicited a greater placebo effect than verbal suggestion alone. Therefore, we hypothesized that observational learning will reinforce the open-label placebo effect (H2).

In addition, we aimed to determine whether the magnitude of the deceptive placebo effect induced by observational learning was comparable with the open-label placebo effect reinforced with observational learning (Q1). Some studies have shown that the open-label placebo effect is similar to the deceptive placebo effect when induced by verbal suggestion or classical conditioning.^19,38,40^

## 2. Materials and methods

### 2.1. Study design

**Figure 1.**
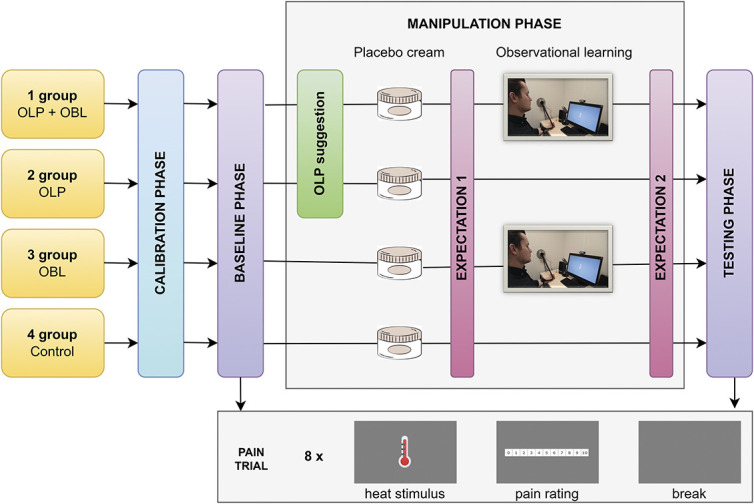
Study design. Participants from the experimental groups underwent 4 phases of the experiment: calibration, baseline, manipulation, and testing. During the manipulation phase, the OLP + OBL and OLP groups received honest information concerning the open-label placebo. Moreover, the OLP + OBL and OBL groups watched a video recording showing a person experiencing hypoalgesia because of the placebo cream application. There was no manipulation phase in the control group. Eight heat pain trials were administered to all participants in both the baseline and testing phases. OBL, observational learning; OLP, open-label placebo.

The study consisted of 4 groups: (1) the open-label placebo effect induced by verbal suggestion and observational learning (OLP + OBL), (2) the open-label placebo effect induced by verbal suggestion alone (OLP), (3) the deceptive placebo effect induced by observation alone (OBL), and (4) a control group. The experiment consisted of 4 consecutive phases: calibration, baseline, manipulation, and testing. In the baseline and testing phases, participants were exposed to a series of thermal stimuli at the same moderate intensities, which were established individually for each participant in the calibration phase. They rated the intensity of each pain stimulus on a numeric rating scale (NRS). During the manipulation phase, the open-label placebo groups (OLP + OBL and OLP) received a placebo cream preceded by the truthful information that the obtained substance was inert. Moreover, participants from the OLP + OBL group watched a video recording of a model who, like the participants, rated pain on the NRS in 2 phases separated by the application of the placebo cream. The pain ratings of the model after the placebo cream application were lower than those before the placebo cream application. In the OBL group, participants received a placebo cream with no information about its effect, and then they watched the same video recording. The placebo cream was applied between the baseline and testing phases in the control group to control for its physical barrier effects. Moreover, participants in all groups rated the expected pain intensity after the application of the placebo cream and after the video presentation (or after a break) (Fig. 1).

### 2.2. Participants

The sample size was determined through an a priori power analysis using G*Power^22^ based on the F test for repeated-measures analysis of variance (ANOVA) and within-between interactions. The expected effect size was estimated based on the results of previous research.^38^ Comparisons among experimental groups have shown that a small-to-medium effect is expected. Therefore, the following values were used to estimate the sample size: effect size f = 0.20, alpha = 0.05, power = 0.80, and correlation among repeated measures = 0.30. The calculation indicated a required sample size of 100 participants (25 in each group). However, to account for potential dropouts and to increase the power to detect the effect, a total of 120 participants were recruited (30 for each group).

The participants were recruited through advertisements on social media and advertising websites. They had to be physically and mentally healthy, aged between 18 and 50 years, free of pain, and not taking any pain medication. The exclusion criteria were prolonged acute pain in the past month; use of illegal drugs; use of alcohol within the past 24 hours; history of any respiratory, circulatory, neurological, musculoskeletal or psychiatric disorders; current symptoms of depression or anxiety; pregnancy; or previous participation in pain-related studies.

Participants were informed that the study aimed to assess how people respond to painful thermal stimulation under different conditions. Having read the description of the experimental procedures, participants provided written informed consent to participate in the study. They were also informed that they could withdraw their consent at any time without providing a reason. When the study was completed, all participants were fully debriefed and informed about the actual aim of the experiment. The participants received 30 Polish złoty (PLN; ∼€7) as compensation for participating in the study. The study protocol was approved by the Research Ethics Committee of the Institute of Psychology, Jagiellonian University, Kraków, Poland (reference number: KE/40_2021).

### 2.3. Apparatus and materials

#### 2.3.1. Pain induction

Thermal heat pain was delivered using the Pathway Pain & Sensory Evaluation System (model ATS, Medoc, Israel). The Pathway System is a commonly used and safe device to induce pain in the experimental setting. To prevent physical injuries, the stimulation stops automatically at a maximum temperature of 51°C.

#### 2.3.2. Placebo cream

The placebo cream that was applied to the participants’ nondominant forearm consisted of a mixture of a standard base cream and a few drops of thyme oil to yield a distinct medicinal scent.

#### 2.3.3. Video recording

The video recording was presented in the manipulation phase of the study in the OLP + OBL and OBL groups. It showed a male model sitting in the same room and in the same position as the participants in front of the computer screen. Both the model seen in profile and the computer screen were visible in the film frame. The model rated thermal pain stimuli administered before and after the application of the placebo cream on the NRS. Pain intensity reported by the model in the first phase ranged from 4 to 6 on the NRS. Pain intensity reported by the model after cream application ranged from 1 to 3 on the NRS. Previous research has shown that male models induce stronger nocebo hyperalgesia than female models, regardless of participant sex^44^; thus, only a male model was used in this study. Observational learning was introduced through video recording, based on previous work by Hunter et al.,^25^ which showed that modeling the placebo effect is similarly effective whether introduced in-person or on video. The use of video recording also allowed to maintain standardized conditions, the same for all study participants.

### 2.4. Measures

#### 2.4.1. Pain and expectations

Pain intensity and pain expectation ratings were obtained by means of an 11-point NRS, ranging from 0 = “no pain” to 10 = “the most intense pain that is tolerable.”

#### 2.4.2. Psychological traits

The following questionnaires were administered to probe the relationship between the magnitude of the placebo effect and the level of empathy:

(1) The *Interpersonal Reactivity Index* (IRI)^18^ is a scale for measuring trait empathy. The Polish version of the questionnaire^30^ contains 21 items divided into 3 subscales: *empathic concern*, *perspective-taking*, and *personal distress.* The items are rated on a 5-point Likert scale.
(2) The *Empathy Quotient* (EQ-Short^51^; Polish adaptation^26^) is a scale designed to measure cognitive and affective empathy. The scale consists of 22 statements that describe the participant's capacity to empathize with other people. The items are rated on a 4-point Likert scale.

#### 2.4.3. Exit questionnaire

At the end of the experiment, the participants were asked to answer the following manipulation check questions (MCQs) to verify the effectiveness of the experimental manipulation and to control for demand characteristics:

(1) What do you think the study you just completed was about?
(2) Did the applied cream affect your sense of pain? If so, what was its effect?
(3) For the OLP + OBL and OBL groups: Did the cream affect the person in the video recording? If so, what was its effect?

### 2.5. Procedure

#### 2.5.1. Calibration phase

Calibration consisted of testing a range of temperatures in ascending order with temperatures starting at 43°C and increasing by 0.5°C with each subsequent heat stimulus (with 5 seconds breaks between pulses). Each stimulus started from a 32°C baseline temperature with a 10°C/second increase and decrease rate and a 3-seconds peak duration. The thermal stimuli were delivered to the ventral surface of the participants' nondominant forearm. Participants rated each of the stimuli aloud using the NRS. Application ended when the participant rated the stimulus as 7 or more on the NRS or when the stimulus reached a temperature of 51°C. Then, the second ascending series was applied in the same manner. Participants' pain ratings from this phase were used to determine individual intensity of pain stimulus corresponding to 4, 5, and 6 on the NRS by fitting them to an exponential curve.

#### 2.5.2. Baseline phase

During the baseline phase, the 8 heat stimuli at the temperatures estimated as 4, 5, or 6 on the NRS were randomly applied to the participants (always with 2 × 4, 4 × 5, and 2 × 6 ratios). Other parameters of thermal stimuli were the same as in the calibration phase. The participants provided pain ratings aloud immediately after experiencing each thermal stimulus.

#### 2.5.3. Manipulation phase

The manipulation phase consisted of 3 distinctive parts: verbal suggestion containing information about the placebo (for the OLP + OBL and OLP groups), application of the placebo cream (all groups), and observation of a person on the video recording (for the OLP + OBL and OBL groups). Parts not administered to some groups were replaced with a correspondingly long break. Moreover, pain expectations were measured twice: once immediately after the placebo cream application and the second time after the video display (or respective break; see Fig. 1).

##### 2.5.3.1. Verbal suggestion

Participants from the OLP + OBL and OLP groups were informed that before the second part of the experiment, they would receive a placebo cream that contained no active medication. Moreover, modified instructions from Kaptchuk et al.^29^ were implemented to familiarize the participants with the placebo effect and its mechanisms. The following bullet points were covered:

1. The placebo effect is powerful. Explanation of what the placebo effect is. Description of research findings on placebo-induced analgesia, such as “it is well known that placebos are very effective, particularly for treating pain, Parkinson disease, depression, migraine, and asthma.”
2. The placebo effect may occur through different psychological mechanisms. Brief description of verbal suggestion, classical conditioning, and observational learning as methods to induce placebo effects. Explanation that the body may respond to a placebo according to expectations or automatically through previous experience.
3. Placebos may work despite participants' awareness that it does not contain pharmacologically active ingredients.

The OBL and control groups were informed that before the second part of the experiment, they would receive a cream. However, the cream's effect was left unexplained.

##### 2.5.3.2. Application of the placebo cream

Placebo cream was applied to the place where the baseline stimulation had previously been administered in all groups.

##### 2.5.3.3. Observation

Participants in the OLP + OBL and OBL groups were asked to watch a video recording along with the following information: “Now, you will be presented with a video featuring a person who went through the same experimental procedure. The recording shows the part of the study you have just finished, as well as the part following the application of the ointment, which you are about to undergo shortly. Please watch the video carefully while the ointment is absorbing.”

While the information mentioned earlier was the same for both OLP + OBL and OBL groups, we assume that it conveyed different meanings depending on the group. In the OBL group, where no information concerning the cream was provided, participants could attribute analgesic properties to the substance by observing alleged participant reactions. This procedure is parallel to other methods that elicit deceptive placebo effects through observational learning without inclusion of verbal suggestion.^5,7,10,44^ In the OLP + OBL group, participants were informed before the video presentation that the cream was inert. They were also explained that observation is one of the methods for inducing the effect. Consequently, participants watched the video with the assumption that the model’s cream was also inactive.

#### 2.5.4. Testing phase

The testing phase was performed in all groups in the same manner as the baseline phase. Afterward, participants completed the IRI and EQ-short and answered manipulation check questions.

### 2.6. Statistical analysis

Of the 120 people recruited for the study, 117 were included in the analyses. Three participants were excluded because of incomplete data (1 person from the OBL group) or the inability to establish safe heat intensities due to participants' very high pain thresholds (above 50°C; 1 participant each from the OLP + OBL and control groups).

Descriptive statistics were calculated for the following variables: age, body mass index (BMI) (mean values and SD values), sex, education level, and employment (percentage and number of participants). To investigate whether there were any between-group differences in these variables, 1-way ANOVA was performed on age and BMI and the χ^2^ test was performed for education level, employment, and sex.

The main analysis was performed on pain intensity ratings using 1-way analysis of covariance (ANCOVA), with group (OLP + OBL, OLP, OBL, or control) as a between-subject factor and the difference between baseline and testing as a dependent variable, while controlling for baseline pain ratings. Analysis of covariance was followed by 1-tailed planned comparison tests. To check whether the placebo effect was successfully induced, each experimental group was compared with the control group in terms of the difference in pain ratings between baseline and testing (H1). Moreover, to verify whether observational learning reinforced the open-label placebo effect, the OLP + OBL and OLP groups were compared in terms of the difference in pain ratings between baseline and testing (H2). In addition, a comparison was conducted between the OLP + OBL and OBL groups regarding the same difference, while applying the Bonferroni correction, to assess potential differences in open-label and deceptive placebo effects induced by observational learning (Q1).

Then, a 1-way ANOVA was performed with group (OLP + OBL, OLP, OBL, or control) as a between-subject factor and the difference between the first and the second expectancy measurements as a dependent variable. Analysis of variance was followed by post hoc tests with Bonferroni correction to explore whether and how pain expectations changed due to viewing the video.

In addition, an analysis was made to explore how expectations could change due to the open-label suggestion. Regrettably, no expectations were assessed before the experimental manipulation. However, to gain at least a preliminary understanding of what might have transpired during the procedure, the 1-way ANCOVA with group as a between-subject factor, the difference between the baseline and the first expectancy measurement as a dependent variable, and the baseline as a covariate was conducted. Analysis of covariance was followed by post hoc tests with Bonferroni correction.

Correlation analysis was performed to explore the relationship between empathy (IRI and EQ-short scores) and the placebo effect (the difference between baseline and testing pain ratings) in the OBL and OLP + OBL groups. In addition, the relationship between pain expectations (the second pain expectation rating) and the placebo effect (the difference between baseline and testing pain ratings) was verified in all groups together.

Finally, the manipulation check answers were examined regarding their ability to influence the main results. The main analysis was performed again after excluding participants who determined the real aim of the study to verify whether it influenced the results.

The alpha level for rejection of the null hypothesis was set at 0.05. Bonferroni correction was implemented for all exploratory analyses. The analyses were conducted using IBM SPSS version 26.0.

## 3. Results

The groups did not differ in terms of participants' age (*F*(3, 113) = 1.30, *P* = 0.277), BMI (*F*(3, 113) = 0.259, *P* = 0.855), sex (χ^2^(3, N = 117) = 0.37, *P* = 0.946), education level (χ^2^(3, N = 117) = 2.34, *P* = 0.886), or employment (χ^2^(3, N = 117) = 11.01, *P* = 0.088). The descriptive statistics and distributions of the participants are summarized in Table 1.

**Table 1:** Descriptive statistics of the participants (data are presented as the mean (±SD) or percentage (number)).

	OLP + OBL (N = 29)	OLP (N = 30)	OBL (N = 29)	Control (N = 29)	All (N = 117)
Age (y)	23.00 ± 5.11	21.60 ± 2.63	23.55 ± 6.25	24.17 ± 6.17	23.07 ± 5.25
BMI (kg/m^2^)	22.92 ± 4.90	22.44 ± 5.37	23.08 ± 4.13	23.56 ± 5.24	23.00 ± 4.89
Sex					
Male	27.6% (8)	33.3% (10)	31.0% (9)	34.5% (10)	31.6% (37)
Female	72.4% (21)	66.7% (20)	69.0% (20)	65.5% (19)	68.4% (80)
Education					
Primary	6.9% (2)	3.3% (1)	3.4% (1)	10.3% (3)	6.0% (7)
Secondary	70.0% (20)	76.7% (23)	79.3% (23)	72.4% (21)	74.4% (87)
Faculty	24.1% (7)	20.0% (6)	17.2% (5)	17.2% (5)	19.7% (23)
Job situation					
Student	82.8% (24)	83.3% (25)	58.6% (17)	62.1% (18)	71.8% (84)
Employed	6.9% (2)	10% (3)	31.0% (9)	17.2% (5)	16.2% (19)
Unemployed	10.3% (3)	6.7% (2)	31.0% (3)	20.7% (6)	12.0% (14)

BMI, body mass index; OBL, observational learning; OLP, open-label placebo.

### 3.1. Main analyses

**Figure 2.**
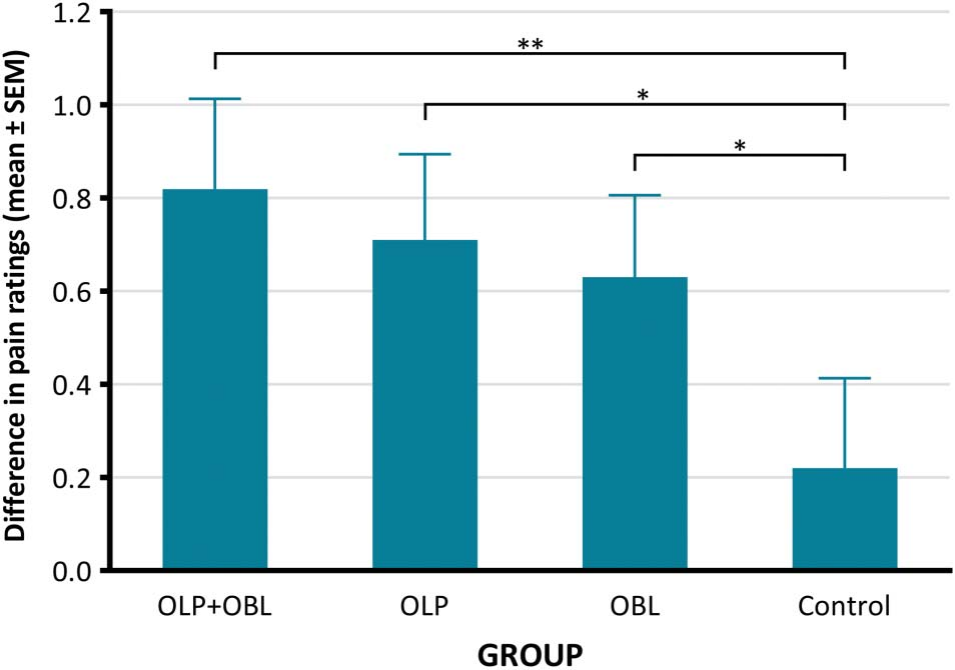
Mean values and standard errors of the differences in pain intensity ratings between the baseline and testing phases for each study group. *P < 0.05, **≤0.01, ***≤0.001. OBL, observational learning; OLP, open-label placebo.

The 1-way ANCOVA revealed that the main effect of group was not significant (*F*(3, 112) = 1.97, *P* = 0.123, ${\text{η}}_{\text{p}}^{2}$ = 0.05). The planned comparison tests showed that the control group differed from the OLP + OBL (*F*(1, 112) = 5.16, *P* = 0.013, ${\text{η}}_{\text{p}}^{2}$ = 0.04), OLP (*F*(1, 112) = 2.99, *P* = 0.044, ${\text{η}}_{\text{p}}^{2}$ = 0.03), and OBL groups (*F*(1, 112) = 3.10, *P =* 0.041, ${\text{η}}_{\text{p}}^{2}$ = 0.03) in terms of the difference between baseline and testing pain ratings. These results indicate that the placebo effect was successfully induced in the OLP + OBL, OLP, and OBL groups (H1). Furthermore, there was no difference between the OLP + OBL and OLP groups (*F*(1, 112) = 0.31, *P* = 0.288, ${\text{η}}_{\text{p}}^{2}$ < 0.01) in baseline and testing ratings, which indicates that observational learning did not significantly reinforce the open-label placebo effect (H2). In addition, there was no difference between the OLP + OBL and OBL groups in this respect (*P* = 1.000), suggesting that open-label and deceptive placebo effects induced by observational learning seem to be of similar strength (Q1) (Fig. 2). Descriptive statistics of the pain ratings are summarized in Table 2.

**Table 2:** Descriptive statistics for baseline and testing pain ratings, as well as for the difference between these scores, in each group and in total (mean ± SD).

	OLP + OBL (N = 29)	OLP (N = 30)	OBL (N = 29)	Control (N = 29)	All (N = 117)
Pain ratings (NRS)					
Baseline	4.91 ± 1.35	5.03 ± 1.09	4.68 ± 1.61	4.81 ± 1.44	4.86 ± 1.37
Testing	4.09 ± 1.34	4.32 ± 1.25	4.05 ± 1.43	4.59 ± 1.58	4.26 ± 1.40
Baseline vs Testing difference	0.82 ± 1.06	0.71 ± 1.03	0.63 ± 0.96	0.22 ± 1.06	0.59 ± 1.04

NRS, numeric rating scale; OBL, observational learning; OLP, open-label placebo.

### 3.2. Exploratory analyses

**Figure 3.**
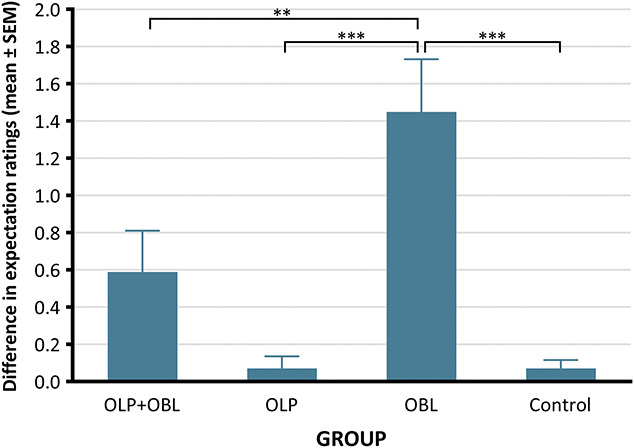
Mean values and standard errors of the differences between the 2 pain expectation measurements in each study group. *P < 0.05, **≤0.01, ***≤0.001. OBL, observational learning; OLP, open-label placebo.

The 1-way ANOVA on pain expectations showed a significant main effect of group (*F*(3, 113) = 12.28, *P* < 0.001, ${\text{η}}_{\text{p}}^{2}$ = 0.25). Therefore, post hoc tests were performed to explore which groups contributed to this effect. The results revealed that the change in expectancy was significantly greater in the OBL group than in the OLP + OBL (*P* = 0.009), OLP (*P* < 0.001), and control groups (*P* < 0.001) (Fig. 3).

The 1-way ANCOVA on the difference between baseline and the first pain expectations showed a significant main effect of group (*F*(3, 112) = 5.70, *P* = 0.001, ${\text{η}}_{\text{p}}^{2}$ = 0.13), while controlling for the baseline. The post hoc tests with Bonferroni correction revealed that the change between baseline and the first pain expectations was significantly greater in the OLP + OBL group (*P* = 0.006) and OLP group (*P* = 0.002) compared with that in the control group.

The correlation analysis showed that there was no relationship between empathy (measured by IRI and EQ-short scores) or the second pain expectations and the magnitude of the placebo effect (Table 3). The manipulation check questions revealed that 27.6% of the OBL group (deceptive placebo) determined the real aim of the study (MCQ1). The main analyses were conducted again after excluding those participants; guessing the real role of the experimental manipulation did not affect the results. Moreover, in all groups, a similar number of participants declared that the applied cream affected their pain sensations (MCQ2; 24.1% in the OLP + OBL group, 40% in the OLP group, 41.4% in the OBL group, and 41.4% in the control group). All participants in the OLP + OBL and OBL groups correctly reported how the pain sensations of the model on the video recording changed after the application of the cream (MCQ3).

**Table 3:** Correlations of empathy (measured by interpersonal reactivity index and empathy quotient-short scores) and the second expectancy measurement with the magnitude of the placebo effect.

	OLP + OBL	OLP	OBL	Control
	Placebo effect			
IRI (EC)	*r* = −0.14; *P* = 0.463	—	*r* = −0.15; *P* = 0.426	—
IRI (PT)	*r* = 0.23; *P* = 0.230	—	*r* = 0.10; *P* = 0.604	—
IRI (PD)	*r* = −0.30; *P* = 0.112	—	*r* = −0.01; *P* = 0.967	—
EQ-short	*r* = 0.27; *P* = 0.161	—	*r* = −0.07; *P* = 0.725	—
Expectations	*r* = −0.08; *P* = 0.387			

Placebo effect = the difference between baseline and testing pain ratings.

EC, empathic concern; EQ-short, The Empathy Quotient; IRI, Interpersonal Reactivity Index; OBL, observational learning; OLP, open-label placebo; PD, personal distress; PT, perspective taking.

## 4. Discussion

Placebo hypoalgesia was successfully induced in all groups with experimental manipulation (OLP + OBL, OLP, and OBL), thus confirming H1. These results are in line with previous research showing that the nondeceptive placebo effect may alter pain experience.^12,38,40^ Therefore, our study provides further evidence that open-label placebo may significantly diminish pain sensation. In addition, it further confirms that placebo hypoalgesia may be successfully induced by observational learning, while using videotaped model.^7,10,25,39^

Furthermore, the placebo effects evoked in groups with the open-label suggestion (OLP + OBL and OLP) were of similar magnitude, regardless of the inclusion of video recordings. Therefore, it seems that observational learning did not facilitate the open-label placebo effect, and H2 was not supported. Previous research on observationally induced deceptive placebo hypoalgesia have shown that adding observation to the suggestion led to a significantly larger effect.^15,25^ However, it seems that these findings do not translate to nondeceptive placebo. By contrast, it seems that for open-label placebo, verbal suggestion itself was responsible for most of the effect. The analysis of expectations delivers an interesting insight on that matter. The expectations were measured immediately before and immediately after the display of the video recording, which enabled precise assessment of how expectations changed under the influence of modeling. The results showed that the change in expectations in the OBL group was significantly larger than that in any other group, including the OLP + OBL group. Moreover, the change in expectations in the OLP + OBL group did not differ in magnitude from that of the OLP and the control group, where only a break separated the 2 measurements. This pattern of results suggests that observation itself influenced participants to a lesser extent when it was preceded by a verbal information. As mentioned earlier, the same video recording could have different reception in the OLP + OBL than in the OBL group. In the OLP + OBL group, participants were informed that the ointment was not active and that the observation of others may help to induce placebo effects. Therefore, the video served as further validation of previously received verbal information. In the OBL group, no information was delivered to the participants, and therefore, they may have tried to figure out the meaning of the movie by themselves.

The results of the correlation analysis confirmed that there was no relationship between the second pain expectations and the magnitude of the placebo effect. Bajcar and Bąbel's model,^3^ as well as Colloca and Miller's model,^17^ postulate that expectancies are central to the formation of placebo effects induced by social observational learning, as previously highlighted by Kirsch.^32^ However, only 1 previous study on observational learning found a correlation between expectations and pain ratings,^4^ while other studies did not find such a connection.^39^ Therefore, it seems that the expectations induced by social information are rarely connected to the magnitude of the placebo effects, and their role requires further investigation. Other correlational analyses revealed that empathy did not contribute to the magnitude of the placebo effect, which is in line with some previous findings^10,45^ but not all of them.^15,25,39^ Hence, the role of observer empathy in inducing the placebo effect is not clear, and further research is needed on this topic.

The additional analysis exploring how expectations may have changed due to the open-label suggestion seems to indicate that the open-label placebo suggestion in the OLP + OBL and OLP groups may have prevented the natural increase in expectations observed in the OBL and control groups. Although the expectations in the OLP + OBL and OLP groups were not enhanced by open-label suggestion, significant placebo hypoalgesia occurred. This finding aligns with previous research indicating that open-label placebo effects are not contingent on the development of positive expectations.^35,42^ However, it should be noted that these results could be significantly affected by the comparison of different variables (pain and expectations) and should be interpreted with caution.

The magnitude of the deceptive placebo effect induced by observational learning did not differ from the open-label placebo effect reinforced with observational learning (Q1). This result is in line with the findings from the previous studies on verbal suggestion or classical conditioning.^19,38,40^ Therefore, it seems that open and deceptive placebo effects are similar in size regardless of the induction methods. However, because this question was covered by an exploratory analysis, more studies need to be conducted to confirm these results.

Moreover, the answers to the manipulation check questions provided an interesting insight. In all groups, a comparable number of participants stated that the cream affected their perceived pain (MCQ2; 24.1% in the OLP + OBL group, 40% in the OLP group, 41.4% in the OBL group, and 41.4% in the control group). Notably, the ratio in the control group was not lower than that in the other groups. It is possible that participants habituated during the experiment and therefore found pain stimuli in the second part of the experiment to be less intense, which they attributed to the cream. In addition, the application of the cream without any additional information might have evoked a classical conditioning mechanism. During daily life, people acquire the experience linked to the topical use of analgesic ointments. It is probable that some of the participants had unintended expectations connected to their previous experiences. This phenomenon was believed to be observed in studies that used medically connoted placebos (eg, cream, pills) because participants might have been preconditioned by previous experiences with similar medical substances or devices.^1,11,39^ Furthermore, MCQ3 revealed that 24.1% of participants in the OBL group guessed the real aim of displaying the video. Sensitivity analysis was conducted to determine whether this knowledge influenced the results. However, no change in the pattern of results was detected.

This study has some strengths that should be acknowledged. First, this is the first study to examine the important learning phenomenon, observation, among open-label placebo studies. Observational learning evoked or facilitated placebo effects in numerous previous studies on deceptive placebos. Therefore, it seems crucial to explore its influence in open-label placebo studies, which have shown increasing promise for use in clinical practice. Moreover, the design of our experiment aimed to minimize difficulties in the interpretation of the results by including a baseline pain measurement, which is somewhat rare in prior research on observational learning.^39^ The lack of comparison with baseline (or any other nonplacebo cue) in those studies often complicated the estimation of whether the pain modulation was ultimately a nocebo effect or a placebo effect. Another advantage of our design was the inclusion of an adequate control group that controlled for natural changes in pain over time, considering the effect of the cream application itself. Open-label studies conducted in clinical settings often lack adequate placebo controls.^8^

Our study also has some limitations, starting with limited translatability into clinical studies. This experiment was conducted on healthy participants and in laboratory settings; therefore, additional research in clinical settings would be advisable before applying the conclusions to other fields. Moreover, the lack of the expectations measurement at the very beginning of the experimental manipulation made it difficult to catch all the expectation fluctuations throughout the study.

## 5. Conclusions

This study provides further evidence that the open-label placebo effect can alleviate pain in healthy participants. Therefore, it contributes to a better understanding of nondeceptive placebo mechanisms and to future clinical management of acute pain. In addition, it shows again that observational learning can induce placebo hypoalgesia. However, observational learning did not seem to reinforce the open-label placebo effect. This outcome may be an important cue regarding the implementation of nondeceptive placebos in clinical practice because it indicates that additional procedures aimed at reinforcing verbal information may be redundant. Nevertheless, this result should be treated with caution, and more studies on this topic are necessary to confirm or contradict it. In addition, more research is needed to determine whether observational learning would induce similar outcomes when used in the clinical setting with patients with chronic pain.

## Conflict of interest statement

The authors have no conflict of interest to declare.
